# Genetic variation in *Staphylococcus aureus *surface and immune evasion genes is lineage associated: implications for vaccine design and host-pathogen interactions

**DOI:** 10.1186/1471-2180-10-173

**Published:** 2010-06-15

**Authors:** Alex J McCarthy, Jodi A Lindsay

**Affiliations:** 1 Centre for Infection, Department of Cellular and Molecular Medicine, St George's, University of London, Cranmer Terrace, London SW17 0RE, UK

## Abstract

**Background:**

*S. aureus *is a coloniser and pathogen of humans and mammals. Whole genome sequences of 58 strains of *S. aureus *in the public domain and data from multi-strain microarrays were compared to assess variation in the sequence of proteins known or putatively interacting with host.

**Results:**

These included 24 surface proteins implicated in adhesion (ClfA, ClfB, Cna, Eap, Ebh, EbpS, FnBPA, FnBPB, IsaB, IsdA, IsdB, IsdH, SasB, SasC, SasD, SasF, SasG, SasH, SasK, SdrC, SdrD, SdrE, Spa and SraP) and 13 secreted proteins implicated in immune response evasion (Coa, Ecb, Efb, Emp, EsaC, EsxA, EssC, FLIPr, FLIPr like, Sbi, SCIN-B, SCIN-C, VWbp) located on the stable core genome. Many surface protein genes were missing or truncated, unlike immune evasion genes, and several distinct variants were identified. Domain variants were lineage specific. Unrelated lineages often possess the same sequence variant domains proving that horizontal transfer and recombination has contributed to their evolution. Surprisingly, sequenced strains from four animal *S. aureus *strains had surface and immune evasion proteins remarkably similar to those found in human strains, yet putative targets of these proteins vary substantially between different hosts. This suggests these proteins are not essential for virulence. However, the most variant protein domains were the putative functional regions and there is biological evidence that variants can be functional, arguing they do play a role.

**Conclusion:**

Surface and immune evasion genes are candidates for *S. aureus *vaccines, and their distribution and functionality is key. Vaccines should contain cocktails of antigens representing all variants or they will not protect against naturally occurring *S. aureus *populations.

## Background

*Staphylococcus aureus *is a highly adaptive and versatile gram-positive bacterium that has major importance to human and animal health. In humans 20% of a healthy population are persistently colonised in the anterior nares of the nose and a further 60% are intermittently colonised [1]. *S. aureus *is a common cause of minor skin and wound infections, but can cause serious and even fatal infections, particularly in the immunocompromised. The emergence of methicillin-resistant *S. aureus *(MRSA) worldwide is of major concern as this dramatically reduces the choice of effective antibiotics for prevention and treatment of a very common infection in both hospitals and communities [2].

*S. aureus *also colonises a range of mammals, including companion animals such as dogs, cats and horses, and livestock such as cows, pigs and goats. It can also colonise birds such as chickens and turkeys. All of these animal species can become infected with *S. aureus*, much like humans, and *S. aureus *is a common cause of dairy cow mastitis with substantial economic impact. Of further concern is the presence of MRSA strains in a variety of animals such as cats, dogs, horses, cows, pigs, chickens and rats [3-7]. These animals may act as important reservoirs for human colonisation as is the case for MRSA sequence type (ST)398 that colonises pigs. Understanding the roles of ecological, epidemiological and genetic factors, and specifically the host- pathogen molecular interactions, involved in host-to-host transmission and colonisation is essential for us to expose novel opportunities for the control of the pathogen. In particular, vaccines for preventing *S. aureus *infection in livestock and/or humans would be useful, but commercial livestock vaccines and human clinical trails have so far proved disappointing.

Adherence is an essential step required for bacterial colonisation of a new host. *S. aureus *can express a variety of surface bound proteins (also referred to as microbial surface components recognising adhesive matrix molecules, or MSCRAMMs) that interact with host extracellular ligands, such as collagen, fibrinogen (FG), fibronectin (FN-1), vitronectin (VN), elastin (ELN), prothrombin (PT) and von Willebrand factor (vWF) [8,9]. In addition, *S. aureus *produce a variety of secreted proteins involved in immune evasion or modulation, often targeting complement and neutrophil recruitment [10-12].

*S. aureus *populations consist of dominant lineages with some minor lineages. Multi- strain whole genome *S. aureus *microarray studies have shown that each *S. aureus *lineage is highly distinct, and that each lineage possesses a unique combination of conserved surface proteins and their regulators [13]. Difference also exists in the expression and secretion of *S. aureus *proteins [14]. The major human lineages are clonal complex (CC)1, CC5, CC8, CC9, CC12, CC15, CC22, CC25, CC30, CC45 and CC51 [15]. The lineages that have acquired *mecA *to become widespread hospital acquired (HA-)MRSA are CC5, CC8, CC22, CC30, CC45 and a hybrid lineage CC239 [16,17]. The lineages that have acquired *mecA *to become widespread community associated (CA-)MRSA are CC1, CC8, CC30, CC59 and CC80 [18]. Companion animals are usually colonised and infected with lineages typically seen in humans [4]. Cows are colonised and infected with their own different lineages that are rarely if ever found in humans, such as CC151, CC771, CC188, CC97, CC130 [14]. In contrast, pigs can be colonised (but are rarely infected) with CC398, which has acquired *mecA*, and this lineage is capable of causing infection in humans [18,19]. Poultry are susceptible to infection with CC5 isolates [20]. Furthermore, there are known to be wide variations in the distribution of lineages between different geographical locations [21,22].

A bounty of new *S. aureus *genome sequences has recently been released into the public domain. Our overall aim was to investigate genetic variation in *S. aureus *core and lineage-specific surface and immune evasion proteins compared to their cognate host proteins, to better identify which are the most likely to be essential during colonisation and infection. We compared whole genome sequences of the first 58 *S. aureus *genomes from 15 lineages and including 4 animal strains. We also extend our previous microarray analysis of human and animals isolates to include human MRSA lineages CC239, CC59 and CC80, and the pig MRSA clone CC398. Since our previous study, a number of new adhesion and immune evasion genes have been characterised, and these are also included in the analysis. Finally, we compared the known and putative human and animal protein targets that interact with *S. aureus *for genetic variation.

## Results

Genes selected for sequence variation analysis fitted one of three criteria; genes that encode (i) surfaced bound proteins that possess LPxTG anchoring motifs (ii) proteins known to interact with host extracellular ligands (iii) secreted proteins that are known to have a role in immune evasion/modulation [9,23]. Gene sequences are avilable from a total of a total of 58 *S. aureus *isolates (Table 1). 25 genes encoding surface bound proteins (Additonal file 1 Table S1) and 13 secreted proteins (Additonal file 2 Table S2) were analysed for sequence variation. Abbreviations of *S. aureus *and host genes and proteins are shown in tables 2 and 3.

**Table 1:** Sequenced *Staphylococcus aureus *genomes

Lineage		Strain	Host	Status	GenBank Accession number	Published reference
CC	ST					
1	1	MSSA476*	H	I	BX571857	[48]
	1	MW2*	H	I	BA000033	[49]
	1	TCH70	H	S	NZ_ACHH00000000	http://www.ncbi.nlm.nih.gov

5	5	A5937	H	I	NZ_ACKC00000000	http://www.broadinstitute.org/
	5	A6224	H	I	NZ_ACKE00000000	http://www.broadinstitute.org/
	5	A6300	H	I	NZ_ACKF00000000	http://www.broadinstitute.org/
	5	A8115	H	S	NZ_ACKG00000000	http://www.broadinstitute.org/
	5	A8117	H	S	NZ_ACYO00000000	http://www.broadinstitute.org/
	5	A9719	H	U	NZ_ACKJ00000000	http://www.broadinstitute.org/
	5	A9763	H	U	NZ_ACKK00000000	http://www.broadinstitute.org/
	5	A9781	H	U	NZ_ACKL00000000	http://www.broadinstitute.org/
	5	A9299	H	U	NZ_ACKH00000000	http://www.broadinstitute.org/
	5	A10102	H	U	NZ_ACSO00000000	http://www.broadinstitute.org/
	5	CF-Marseille	H	I	NZ_CABA00000000	[50]
	5	ED98*	A	I	CP001781	[20]
	5	Mu3*	H	I	AP009324	[51]
	5	Mu50*	H	I	BA000017	[52]
	5	N315*	H	S	BA000018	[52]
	105	JH1*	H	I	CP000736	[53]
	105	JH9*	H	I	CP000703	[53]

7	7	USA300 TCH959*	H	S	NZ_AASB00000000	http://www.ncbi.nlm.nih.gov

8	8	A5948	H	U	NZ_ACKD00000000	http://www.broadinstitute.org/
	8	A9765	H	U	NZ_ACSN00000000	http://www.broadinstitute.org/
	8	NCTC 8325*	H	S	CP000253	[54]
	8	Newman*	H	I	AP009351	[55]
	8	USA300 FPR3757*	H	I	CP000255	[56]
	8	USA300 TCH1516*	H	S	CP000730	[57]
	250	COL*	H	S?	CP000046	[58]

10	10	H19	H	U	NZ_ACSS00000000	http://www.broadinstitute.org/
	145	D139	H	U	NZ_ACSR00000000	http://www.broadinstitute.org/

22	22	EMRSA15/5096*	H	I		http://www.sanger.ac.uk/pathogens

30	30	55/2053	H	U	NZ_ACJR00000000	http://www.broadinstitute.org/
	30	58-424	H	U	NZ_ACUT00000000	http://www.broadinstitute.org/
	30	65-1322	H	U	NZ_ACJS00000000	http://www.broadinstitute.org/
	30	68-397	H	U	NZ_ACJT00000000	http://www.broadinstitute.org/
	30	A017934/97	H	U	NZ_ACYP00000000	http://www.broadinstitute.org/
	30	Btn1260	H	U	NZ_ACUU00000000	http://www.broadinstitute.org/
	30	C101	H	U	NZ_ACSP00000000	http://www.broadinstitute.org/
	30	E1410	H	U	NZ_ACJU00000000	http://www.broadinstitute.org/
	30	M1015	H	U	NZ_ACST00000000	http://www.broadinstitute.org/
	30	M876	H	U	NZ_ACJV00000000	http://www.broadinstitute.org/
	30	M899	H	U	NZ_ACSU00000000	http://www.broadinstitute.org/
	30	MN8	H	S	NZ_ACJA00000000	http://www.ncbi.nlm.nih.gov
	30	TCH60	H	S	NZ_ACHC00000000	http://www.ncbi.nlm.nih.gov
	30	WBG10049	H	V	NZ_ACSV00000000	http://www.broadinstitute.org/
	30	WW2703/97	H	U	NZ_ACSW00000000	http://www.broadinstitute.org/
	34	C160	H	U	NZ_ACUV00000000	http://www.broadinstitute.org/
	36	MRSA252*	H	I	BX571856	[48]

42	42	C427	H	U	NZ_ACSQ00000000	http://www.broadinstitute.org/

45	45	A9635	H	U	NZ_ACKI00000000	http://www.broadinstitute.org/

72	72	TCH130	H	S	NZ_ACHD00000000	http://www.ncbi.nlm.nih.gov

151	151	RF122/ET3-1*	B	I	AJ938182	[59]

239	239	JKD6008	H	I	NZ_ABRZ00000000	http://www.ncbi.nlm.nih.gov
	239	JKD6009	H	I	NZ_ABSA00000000	http://www.ncbi.nlm.nih.gov
	239	0582/TW20*	H	I	FN433596	http://www.sanger.ac.uk/pathogens

398	398	S0385 *	P/H	I	AM990992	http://www.ncbi.nlm.nih.gov

425	425	LGA251*	B	I		http://www.sanger.ac.uk/pathogens

431	431	M809	H	U	NZ_ACUS00000000	http://www.broadinstitute.org/

58 *S. aureus *genomes have been sequenced to date from14 different clonal complex lineages (CC). CC5, CC8 and CC30 are the most extensively sequenced with17, 8 and17 genomes currently available. Sequence type (ST) of each strain is shown, and strains with fully annotated genomes are denoted with an (*). Host origin of the strain is denoted as avian (A), bovine (B), human (H) and porcine (P). Infection status of the strain is shown as a strain isolated from an infection (I), a strain isolated from another source (S) or a strain for which the origin is publically unavailable (U). GenBank accession numbers are shown where possible, as well as published references or corresponding websites.

**Table 2:** Abbreviation list of *Staphylococcus aureus *genes/proteins

Protein name	Gene symbol	Protein symbol	Published reference
Autolysin/adhesin from *S. aureus*	*aaa*	Aaa	[60]

Clumping factor A	*clfA*	ClfA	[61-63]

Clumping factor B	*clfB*	ClfB	[64,65]

Chemotaxis inhibitory protein of Staphylococcus *aureus*	*chp*	CHIP	[39]

Collagen adhesin	*cna*	Cna	[66-68]

Coagulase	*coa*	Coa	[69,70]

Extracellular adhesion protein	*eap*	Eap	[71-73]

ECM-binding protein homologue	*ebh*	Ebh	[74-77]

Elastin-binding protein of Staphylococcus aureus	*ebps*	EbpS	[78-80]

Extracellular complement-binding protein	*ecb*	Ecb	[11]

Extracellular fibrinogen-binding protein	*efb*	Efb	[81-83]

Extracellular matrix protein	*emp*	Emp	[84]

ESAT-6 secretion accessory C	*esaC*	EsaC	[85,86]

ESAT-6 like factor A	*esxA*	EsxA	[86]

ESAT-6 secretion system C	*essC*	EssC	[86]

FPR-like1 inhibitory protein	*fll*	FLIPr	[10]

FPR-like1 inhibitory protein like	*flr*	FLIPr-like	[11]

Factor which affects the methicillin resistance level and autolysis in the presence of Triton X-100 B	*fmtB*	FmtB	[24,87]

Fibronectin binding protein A	*fnbpA*	FnBPA	[24,88-93]

Fibronectin binding protein B	*fnbpB*	FnBPB	[89]

Immunodominant surface antigen B	*isaB*	IsaB	[94]

Iron-regulated surface determinants A	*isdA*	IsdA	[9,95-97]

Iron-regulated surface determinants B	*isdB*	IsdB	[9,96]

Iron-regulated surface determinants H	*isdH*	IsdH	[9,97,98]

Penicillin-binding protein 2a conferring resistance to methicillin	*mecA*	MecA	

Staphylokinase	*sak*	SAK	[39]

*Staphylococcus aureus *protein B	*sasB*	SasB	[87]

*Staphylococcus aureus *protein C	*sasC*	SasC	[23,99]

*Staphylococcus aureus *protein D	*sasD*	SasD	[22]

*Staphylococcus aureus *protein F	*sasF*	SasF	[22,100]

*Staphylococcus aureus *protein G	*sasG*	SasG	[22,101,102]

*Staphylococcus aureus *protein H	*sasH*	SasH	[22,103]

*Staphylococcus aureus *protein K	*sasK*	SasK	[22]

*Staphylococcus aureus *binder of IgG	*sbi*	Sbi	[12,104,105]

Staphylococcal complement inhibitor	*scn*	SCIN	[39]

Staphylococcal complement inhibitor B	*scin-B*	SCIN-B	[11]

Staphylococcal complement inhibitor C	*scin-C*	SCIN-C	[11]

Serine aspartate repeat protein C	*sdrC*	SdrC	[106,107]

Serine aspartate repeat protein D	*sdrD*	SdrD	[106-109]

Serine aspartate repeat protein E	*sdrE*	SdrE	[106]

*Staphylococcus aureus *protein A	*spa*	Spa	[110-112]

Serine-rich adhesin for platlets	*sraP*	SraP	[113,114]

von Willebrand factor-binding protein	*vwb*	VWbp	[115-117]

**Table 3:** Abbreviation list of host genes/proteins used in the manuscript

Protein name	Gene symbol	Protein symbol
Complement protein 3		C3

C3 convertase		C4b2a

Collagen type1		CN-1

Cytokeratin10		CK10

Elastin	*emn*	ELN

Fibirogen	*fga, fgb, fgg*	FGA, FGB, FGG

Fibronectin	*fn-1*	FN-1

Formyl peptide receptor		FPR

FPR-Like-1		FPRL1

Haemoglobin		Hb

Haptoglobin		Hp

Immunogblubin G		IgG

Tumour necrosis factor receptor1		TNRF1

Thrombospondin1		TSP-1

Vitronectin	*vtn*	VN

Prothrombin	*pt*	PT

Von Willebrand factor	*vwf*	vWF

### Sequence variation in surface bound S. aureus proteins

Eight (*ebpS*, *fnbpA*, *isaB*, *isdA*, *isdH*, *sasF*, *sasH *and *spa*) of the 25 genes that encode these surface bound proteins were present in all sequenced *S. aureus *genomes. Some of the remaining 16 genes were absent from a small number of *S. aureus *genomes (*clfA*, *clfB*, *eap*, *ebh*, *fnbpB*, *isdB*, *sasB*, *sasC*, *sasD*, *sasG*, *sdrC*, *sdrD *and *sraP*) whilst others were absent from the majority of *S. aureus *genomes (*cna*, *sasK *and *sdrE*) (Additonal file 1 Table S1). This indicates that many surface bound proteins are not essential for survival and replication of *S. aureus*. Many of these proteins are known to adhere to the same host ligand and redundancy in *S. aureus *adhesins is common.

Variation was identified in all 25 surface bound proteins indicating that surface adhesins are not only present/absent, but also variable amongst *S. aureus *genomes. Variation between lineages was higher than within lineages for all genes (Additonal file 1 Table S1). The protein domains at the host-interface had a higher level of interlineage variation in comparison to other protein domains for 12 *S. aureus *surface bound proteins (Aaa, ClfA, ClfB, Eap, EbpS, FnBPA, FnBPB, SasG, SdrC, SdrD, SdrE and Spa) (Additonal file 1 Table S1). The iron-regulated surface adhesins (IsdA, IsdB and IsdH), Cna, SasB and SraP have lower levels of interlineage variation present in putative host-interface domains than other protein domains. Knowledge of protein domains in the remaining 6 proteins is not currently available; nonetheless there are higher levels of interlineage variation in the N terminus of IsaB, SasC and SasF, in comparison to the C terminus.

Intralineage amino acid variation is present in all surface bound proteins. Low levels of variation (proportion of variable sites < 0.05) exist in 22 surface proteins, whilst SdrD, Spa and SraP have higher levels of intralineage variation. Across all proteins there are small levels of intralineage variation in host-interface domains (proportions of variable amino acid sites vary from 0.000 to 0.078) (Additonal file 1 Table S1). Interestingly, intralineage levels of variation differ between lineages in host-interface domains of a small subset of surface bound proteins. For example, the FN-1 binding domain of FnBPA has a proportion of variable amino acid sites of 0.032, 0.016 and 0.008 for CC5, CC8 and CC30 respectively, whilst there is an interlineage variation of 0.139. Such variation could support *S. aureus *lineage adaption to hosts and environments, and/or *S. aureus *evasion of the host immune response.

An example of a highly variable surface protein is FnBPA. The distribution of protein domain variants of FnBPA across CC lineages shows evidence of recombination. (Additonal file 3 Table S3). For the purposes of this paper we define a domain variant as any domain with a sequence encoding one amino acid difference. In addition, we define a domain that has greater than 5% of variable amino acids as a major variant within a domain. The data shows that a range of major and/or minor sequence variations exist for the N terminus of the variable region domain, the fibrinogen (FG) and elastin (ELN) binding domain and the fibronectin (FN-1) binding domain (Additonal file 3 Table S3). Within each CC lineage only one major sequence variant exists for each FnBPA domain, and therefore the whole gene is lineage-specific. Surprisingly, the same major sequence variant of a domain is often found in unrelated lineages. Furthermore, whilst a lineage may share a major sequence variant of one domain with one unrelated lineage, it may share a major sequence variant at an adjacent domain with a different unrelated lineage. This shows that the *fnbpA *gene has a mosaic structure and indicates the *fnbpA *locus is evolving through recombination, in addition to point mutation. Loughman *et al*. [24] have previously identified FnBPA sequence variants from human strains of lineages that have not had their genome sequenced (CC12, CC15, CC25, CC55, CC59, CC101, CC121 and CC509) and classified seven isotypes. They have shown that all isotypes have human fibrinogen binding activity, but that isotype I (found in CC8, CC15 and CC55) binds weakly to elastin. Inclusion of these partial gene sequences [GenBank: AM749006-15], corresponding to amino acid residues 1- 565, in our analysis suggests these gene variants are typical. Interestingly, they prove that no animal *S. aureus *strain has a major domain variant that is not found in a human *S. aureus *lineage. The bovine strain LGA25 1 (CC425) shares a major variant of the FG and ELN binding domain with human strains TCH959 (CC7) and 3110 (CC 12). Likewise, bovine strain RF122 (CC151) has a major variant of the FG and ELN binding domain that is also found in strains D139 and H19 (both CC10). Porcine strain S0385 (CC398) shares a major variant of the FG and ELN binding domain with human strain 3153 (CC509), varying at only 11 amino acid residues. The N terminus of the variable region of these three strains is a recombination of sequences found in a range of human *S. aureus *lineages. This indicates that animal *S. aureus *lineages have domain variants also found in human *S. aureus *lineages.

Interestingly, animal lineages possess a unique combination of FnBPA domain variants that are not found in human lineages (Additonal file 3 Table S3). A unique combination of domain variants is also found in animal isolates in other surface bound proteins (ClfA, Eap, Ebh, EbpS, IsdB, SdrD and SdrE). In addition, novel domain variants are found in animal lineages in other surface bound proteins (FnBPB, IsdA, IsdH and SasB). Interestingly, much of this novel domain variation has been generated by intradomain recombination events. These proteins could be important in the adaptation of *S. aureus *to different host species. Determining whether animal lineages truly have a unique domain variant or possess a unique combination of domain variants can only truly be resolved by future sequencing of other major human *S. aureus *lineages, or through future microarray studies. For other surface proteins, animal lineages do not have a unique combination of domain variants, and neither do they possess unique domain variants (Aaa, ClfB, Cna, IsaB, SasC, SasF, SasG, SasH, SasK, SdrC, Spa and SraP). This therefore questions the importance of these genes in the adaptation of *S. aureus *lineages to different host species.

### Sequence variation in secreted S. aureus proteins

The sequence variation of 13 secreted *S. aureus *genes encoding proteins that have characterised or hypothesised roles in immune evasion was analysed (Additonal file 2 Table S2). Eight (*coa*, *ecb*, *efb*, *emp*, *esxA*, *essC*, *sbi *and *vwbp*) of the 13 secreted genes are present in all sequenced *S. aureus *genomes. In addition, each genome either possesses a gene encoding FLIPr or FLIPr-like and SCIN-B or SCIN-C suggesting that the function of these homologs is essential to *S. aureus *survival and replication (Additonal file 2 Table S2). As functions of all these proteins, except EsaC, are present in all sequenced genome this suggests that secreted proteins involved in immune evasion are critical to *S. aureus*.

All 13 secreted proteins are variable amongst *S. aureus *genomes (Additonal file 2 Table S2). There is a higher level of interlineage variation in host interface domains than other domains for Coa and vWbp. In Efb there is greater variation in the signal sequence than domain characterised in host interactions. Sbi has a characterised host- interface domain, yet there is more variation in the C terminus (proportion of variable residues = 0.25 1) than the Ig or C3 binding domains (proportion of variable residues ranges from 0.000 to 0.125). Functional domains are currently unidentified for Ecb, Emp, EsaC, EsxA, EssC, FLIPr, FLIPr-like, SCIN-B and SCIN-C.

Intralineage variation is present in Coa, Efb, Emp, EssC, FLIPr, Sbi and VWbp at low levels (proportion of variable sites < 0.0 19) and absent in the remaining proteins. The exception is FLIPr-like which is more variable and frequently truncated. The level of and location of intralineage variation differs between the CC5, CC8 and CC30 lineages. The secreted proteins involved in immune evasion of *S. aureus *lineages may be differentially adapted, but that there was little adaptation of strains within lineages.

An example of a highly variable immune evasion gene, *coa *or coagulase, is shown in more detail in additonal file 4 Table S4. There are a variety of conserved domains spread amongst the lineages. Similarly to FnBPA, unrelated lineages often share the same domain variants (Additonal file 4 Table S4). However, there is less evidence of recombination within the *coa *gene than within the *fnbpA *gene as there are fewer examples of unrelated lineages sharing the same sequence variant. An exception to this is the C terminus. The pig CC398 *coa *gene is highly similar to the human CC45 *coa *gene. The avian CC5 strain has the same gene as the human CC5. The bovine CC425 is similar to human CC5 genes but has a different central region, while the bovine CC151 strain has a unique *coa *gene.

Animal lineages possess unique combinations of Coa domain variants that are not found in human lineages, similar to FnBPA (Additonal file 4 Table S4). Animal lineages also have a unique combination of domain variants for other secreted proteins (Emp and VwBP). Animal lineages possess unique domain variants in EssC, SCIN-B and VwBP, whilst for other secreted proteins (Ecb, Efb, EsaC, EsxA, FLIPr, FLIPr-like, SCIN-C and Sbi) animal lineages do not have unique domain variants or a unique combination of domain variants.

### Microarray data

**Figure 1:**
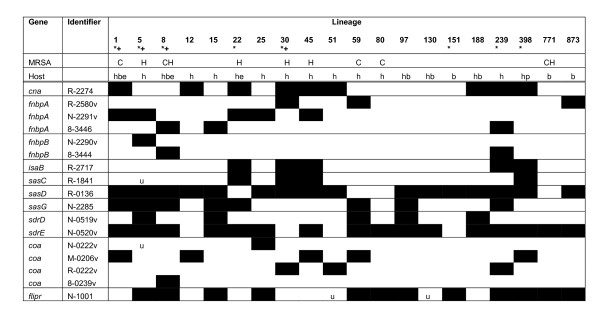
**Microarray analysis**. Microarrays show gene variants are conserved across unrelated lineages. Genes are listed in order by name and by their annotated gene number prefixed with the strain that was used as the template for the PCR probe on the microarray (R, MRSA252; N, N315; 8, 8325; M, MW2; U, Mu50). A black box indicates the gene or gene variant is present in that lineage. '*' indicates the genome of a strain from this lineage has been sequenced. '+' indicates ORFs from this lineage are included on the 7 strain microarray. C indicates community associated MRSA were included, and H indicates hospital associated MRSA were included. Strains from the following hosts were included: h, human, b, bovine, e, equine, p, pig. 'v' denotes a PCR product designed to a specific variant region. 'u' indicates variation in gene distribution for that lineage.

Microarray data is useful for confirming the distribution of genes amongst large populations, for showing that lineages are conserved, and investigating unsequenced lineages. Using the seven-strain *S. aureus *microarray the 400 isolates, representing MSSA, HA-MRSA, CA MRSA and from human, bovine, equine, pig, goat, sheep and camel, clustered into 20 dominant lineages. The distribution of surface and secreted gene variants is shown in Fig. 1, and confirms that all strains of a lineage usually carry the same distribution of surface and immune evasion genes and variants, and that variants are often distributed across unrelated lineages. The distribution of genes with poorly performing spots (*eap*, *ebpS*, *emp*, *esaC*, *scin-c *and *vwbp*) and genes with spots that do not discriminate lineage-specific variation (*clfA*, *clfB*, *ecb*, *efb*, *EsxA*, *essC*, *isdA*, *isdB*, *isdC*, *isdH*, *sasB*, *sasF*, *sasH*, *sasK*, *sbi*, *sdrC*, *spa *and *sraP*) are not reported in this microarray study. Some gene variants are not included in the microarray design as they were not identified in the first seven *S. aureus *whole genome sequencing projects [25].

### Variation in host ligands of S. aureus proteins

The location of and proportion of amino acid variable sites for human ligands are shown in Table 4. Variation is present in each of the ligands analysed. Notably, the proportion of variable residues is high (>0.0 15) in the β-chain (FGB) and γ-chain (FGG) of fibrinogen, and in elastin (ELN). Lower levels of variation exist in the α-chain (FGA) of fibrinogen (0.0 10), promthrombin (PT) (0.006), vitronectin (VN) (0.006), fibronectin (FN-1) (0.006) and the von Willebrand factor (vWF) (0.008). This analysis shows that the amino acid sequence in *S. aureus *ligands varies in humans, and some of this variation is in domains interacting with ClfA, ClfB and FnBPA. This could provide a selective pressure for the evolution and adaptation of *S. aureus *adhesins in human populations.

**Table 4:** Variation in human proteins

Ligand	Gene	NCBIGeneID	Variable amino acid sites	Proportion of variable sites	Characterised interacting *S. aureus *protein(s)
Elastin	*eln*	2006	40, 71,165, 298, 311, 398, 422, 463, 494, 503, 544, 581, 651, 711	0.019	EbpS, FnBPA
Fibrinogen/Fibrin	*fga*	2243	6, **331**^**b**^, 392, 446, 456, 507, 729	0.010	ClfB, FnBPB, Ebh, IsdA,
	*fgb*	2244	2, 86,100,170,192, 265, 398, 478	0.016	Efb,
	*fgg*	2266	12,14, 25, 54, 77, 87, 89,113,114,132,140,177,191, 219, 410^ac^	0.033	ClfA, FnBPA
Fibronectin	*fn-1*	2335	15, **251**^**c**^, 352, 759, 817, 984,1044,1103,1558, 2195, 2212, 2261, 2275, 2281	0.006	FnBPA, FnBPB,
Prothrombin	*f2*	2147	165, 200, 272, 386	0.006	VWbp
Vitronectin	*vtn*	7448	122, 268, 400	0.006	Unknown protein [118]
von Willebrand factor	*vwf*	7450	137, 318, 325^d^, 471, 484, 653, 740, 817, 852, 885,1380,1381,1435,1472,1565,1569, 2126, 2178, 2281, 2342, 2561, 2705	0.008	VWbp Spa

^a ^residues located in ClfA binding region [61].

^b ^residues located in ClfB binding region [64].

^c ^residues located in FnBPA binding region [91,92].

The interspecies similarity of host ligands was calculated for FGB, FGG, FN-1, PT and vWF (Additonal file 5 Tables S5-S9). Species included in analysis are those for which full sequence is available and annotated. For FGA, ELN and VTN there was too much variation amongst interspecies sequences to construct a reliable alignment. Interspecies similarity matrices are therefore not reported for these ligands. For fibrinogen the analysis shows that considerable variation exists in both FGB and FGG between humans and other animal species that become colonised with *S. aureus*, such as dog, cow and horse (Additonal file 5 Tables S5 and S6). Interestingly, FGB (similarity = 79.1%) has a lower similarity score for human and cow homologs than FGG (similarity = 83.7%) revealing that levels of interspecies variation differ between chains of complexes for this species pair. Surprisingly, the animal species that has the lowest identity to human sequence varies amongst the ligands. For example, the similarity of human vWF to that of pig and cow is 0.559 and 0.810 respectively, whilst the similarity of human PT to that of pig and cow is 0.828 and 0.812 respectively (Additonal file 5 Tables S8 and S9). This analysis shows that there is a substantial interspecies variation in host ligands that consequently will provide a selective pressure for the adaptation of *S. aureus *adhesins.

## Discussion

The multitude of sequencing projects available in the last year has confirmed previous observations about *S. aureus *population structure but also revealed some new surprises. In this manuscript we have focussed specifically on those proteins that are predicted to interact with host because of their importance in vaccine development, but also because they are presumed to define the host-pathogen interaction.

Our analysis proves that variation in genes encoding surface proteins is lineage specific, but that many domain variants are conserved across unrelated lineages. Most of the variation occurs in predicted functional domains. Many are missing in some lineages, or are frequently truncated. Similarly, the genes encoding secreted proteins predicted to interact with host immune responses also show variation that is lineage specific, conserved across unrelated lineages, and occurs in predicted functional domains. The amount of variation in immune evasion genes is less than in the surface proteins, and missing or truncated proteins are less common.

The surface proteins are major targets for vaccine development. Vaccines to ClfA, ClfB, FnBPA, IsdA, IsdB, SdrD, SdrE, Eap, Emp have shown protection in animal models as have capsule and haemolysin A [26-32]. The animal model work typically involves vaccinating against one surface protein variant, and then exposing the animals to a challenge strain expressing the same surface protein variant. Human trials of capsule vaccines to prevent infection or colonisation have been disappointing [33,34]. A trial of a vaccine to enhance ClfA antibody produced sera that did not protect low birth-weight babies from sepsis [35]. Furthermore, commercial vaccines for livestock are generally not effective [36]. Yet, *S. aureus *surface proteins are currently in human vaccine trials.

Humans are exposed to a variety of *S. aureus *lineages. This paper clearly shows that *S. aureus *populations carry a range of unique variants of surface proteins. Therefore, animals in vaccine trials should be challenged with a range of *S. aureus *lineages so that the vaccine is tested with a representative range of *S. aureus *surface proteins. If the vaccine is protective against a range of strains, it may then be suitable for human trials. Vaccines cocktails of multiple surface proteins have been tested in animals [27]. However, these also use the variants found in only one laboratory lineage. To obtain good coverage, multiple variants of multiple targets in the vaccine cocktail will likely be more effective. The lack of variant antigens in the vaccines currently tested in animals, humans and livestock may explain their failure to protect from infection with naturally occurring *S. aureus *populations in the non-laboratory environment.

We note that MRSA strains in our collection typically had the same surface and secreted protein profiles as methicillin-sensitive *Staphylococcus aureus *(MSSA) from the same lineage. We did not find a surface or secreted immune marker of MRSA, nor of HA-MRSA or CA-MRSA strains.

If a surface protein is dispensable in some lineages that are still able to cause disease, then its role in virulence is called into question. Many surface proteins appear to bind multiple host proteins, and multiple surface proteins may bind the same host protein [9]. Therefore, the role of individual proteins in disease is difficult to prove and it seems likely that a combination of proteins is essential for virulence. Intriguingly, some lineages are thought to be more associated with particular human hosts than others [37]. We can show there are subtle variations in the genetic sequences of human host proteins, especially in binding regions, which may be implicated in this host specificity.

Unexpectedly, the sequences of the animal lineages of *S. aureus *do not support this hypothesis. If animal strains of *S. aureus *interact with animal host proteins the bacteria would be expected to have animal specific binding proteins or domains. However this is generally not the case, and the animal strains show gene sequences remarkably similar to those found in human strains. No unique surface proteins with an LPxTG anchoring domain could be identified in any of the animal sequencing projects [38]. Yet, the sequence of predicted animal protein targets is substantially different from human counterparts. How do *S. aureus *strains interact specifically with non-human hosts? The importance of individual proteins in host-pathogen interactions is therefore difficult to confirm.

One factor that is not taken into account in this study is the possibility of strains acquiring additional genes on mobile genetic elements (MGEs). Indeed, we have previously shown that a bacteriophage of the phi3 family is found in most human isolates of *S. aureus *but in only about 20% of animal strains [14]. This phage frequently carries genes encoding human specific immune evasion proteins chemotaxis inhibitory protein (*chip*), staphylococcal complement inhibitor (*scin*, (unique from *scin-B *and *scin-C*) and staphylokinase (*sak*) [39]. Our analysis of the animal *S. aureus *strain genome sequences did not identify any novel MGE genes with a possible surface or immune evasion function. Although it is true that novel immune evasion genes can be difficult to identify from sequence alone, and some may be characterised in the future. The distribution of these genes among large populations awaits large scale comparative genomics studies using sequencing or extended microarray platforms.

The fact that surface and immune evasion proteins varied predominantly in predicted functional regions suggests these proteins do play a role in host interaction and that variants have been selected for. Loughman *et al*. [24] have investigated seven variants (isotypes) of the FnBPA protein for their ability to bind human fibrinogen and elastin. All variants bound fibrinogen equally well, but one variant bound elastin less efficiently. The fact that all the variants had activity supports the idea that FnBPA does indeed play a role in host-pathogen interaction as presumably variants that do not bind are not selected for. But it is also interesting that elastin binding could be dispensable. Jongerius *et al*. [11] have shown that SCIN-B and SCIN-C are unable to inhibit AP-mediated hemolysis in serum of species other than humans. They also showed that Ecb and Efb blocked complement of human and 7 other species. Therefore, the function of all variants against all hosts cannot be assumed until appropriate biological studies are performed.

Although human and animal lineages have been well described, some human strains do cause infection in animals and vice versa [4,12,40]. If specific host-pathogen interactions are necessary, then perhaps each strain carries one or more key surface and immune evasion proteins that are specific to each of the animal species they colonise. Alternatively, some bacterial proteins may interact with a broad host range. Biological studies to investigate these hypotheses across a broad range of surface and immune evasion proteins are needed.

While 58 genomes are currently available for analysis, there are still many lineages of *S. aureus *that have not been sequenced. This is likely to change in the next few years. However, our analysis suggests that the majority of genes on the stable core and lineage specific regions of the genome may have been sequenced already, and few very different genes or gene variants will be described. The exceptions may be in *fnbpA *and *coa *which seem to be remarkably variable and frequently recombining. Single nucleotide polymorphisms and variable repeat regions in genes (Additonal file Table S1 and additional 2 Table S2) are also expected to continue to evolve and vary. New genomes may reveal new surprises, and often identify new MGEs [41].

## Conclusions

In summary, the similarity of surface and immune evasion genes in *S. aureus *strains from different animal hosts with very different target proteins is surprising and suggests specific host-pathogen interactions via these proteins are not essential for virulence. However, variation in *S. aureus *proteins is predominantly in predicted functional regions and there is some biological evidence that variant bacterial proteins can have similar functions [24]. This argues that specific host-pathogen interactions of these proteins are essential for virulence. This is an area of research that requires further investigation. Importantly, vaccine development should utilise information on the variation, distribution and function of surface protein antigens amongst lineages to ensure that cocktails of gene variants are included. Otherwise vaccines may fail in human trials, and/or encourage selection of lineages different to those of laboratory strains, including CA-MRSA.

## Methods

### Staphylococcus aureus genomes

Sequence data is available for the genomes of 58 *Staphylococcus aureus *isolates on the GenBank database http://www.ncbi.nlm.nih.gov and the Broad Institute website http://www.broadinstitute.org/. The source and accession numbers of these genomes is shown in table 1. The genetic sequence of an additional 3 *S. aureus *genomes was made available by Matt Holden (EMRSA-15 and LGA251; Sanger Centre, UK) and Ad Fluit (S0385; University Medical Centre Utrecht, Netherlands). Strains are of human origin except strain RF122 which is a bovine mastitis isolate, strain LGA25 1 from a bovine infection, strain ED98 from a diseased broiler chicken, and strain ST398 isolated from a human but likely from pig origin. Sequence analysis was therefore performed on the genomes of 58 *S. aureus *isolates that represent 18 different multi locus sequence types (MLST) (ST1, ST5, ST7, ST8, ST22, ST30, ST34, ST36, ST42, ST45, ST72, ST105, ST145, ST151, ST239, ST250, ST398, ST425 and ST431) and 15 different clonal complex (CC) lineages (CC1, CC5, CC7, CC8, CC10, CC22, CC30, CC42, CC45, CC72, CC151, CC239, CC398, CC425 and CC431) (Table 1). It should be noted that some of the genomes are not complete, and some may have minor errors that lead to the overestimation of truncated proteins.

### Sequence analysis of Staphylococcus aureus genes

The sequence of each gene in a genome was first identified using the BLAST function of the GenBank database http://www.ncbi.nlm.nih.gov/blast. Sequences of a gene were subsequently aligned using the ClustalW program and then edited by hand if necessary in BioEdit [42,43]. Domains of *S. aureus *proteins were identified using the UniProt resource of protein sequence and function http://www.uniprot.org and/or from previous literature. In cases where the domains of a protein have not previously been characterised, the signal sequence was identified using SignalP 3.0 and the remaining sequence was split into an N-terminus and C- terminus [44].

The proportion of variable sites in each protein domain was calculated between all sequences available for each *S. aureus *gene, and is denoted as interlineage variation. The proportion of variable sites within protein domains was also calculated within CC lineages for CC5, CC8 and CC30, as these lineages had genome sequence available from multiple isolates (17, 7 and 18 isolates respectively). Within these CC lineages the extent of intralineage variation was calculated for ST5, ST8 and ST30, respectively. The extent of interlineage and intralineage variation in *S. aureus *proteins involved in adherence and nasal colonisation and/or immune modulation can therefore be compared.

### Microarray analysis

A total of 400 *S. aureus *isolates were analysed representing MSSA, HA-MRSA, CA MRSA and from human, bovine, equine, pig, goat, sheep and camel. The microarray used in this study was developed and comprehensively described previously [12,23]. Data from previous studies and additional strains from St George's Hospital Trust and kindly donated by Mark Enright are included [12,14,40,45-47].

### Sequence analysis of host ligand genes

The sequence of the human genes encoding fibrinogen (FG), fibronectin (FN), elastin (ELN), vitronectin (VN), prothrombin (PT) and von Willebrand factor (vWF) were isolated from the GenBank database, accession numbers are shown in Additonal file 3 Tables S3. Variable sites of each ligand were identified from the GenBank SNP resource http://www.ncbi.nlm.nih.gov/SNP and the proportion of variable sites was calculated.

The sequence of animal genes encoding fibrinogen (FG), fibronectin (FN-1) prothrombin (PT) and von Willebrand factor (vWF) were identified by BLAST search with human gene sequences and aligned in ClustalW program and then edited by hand if necessary in BioEdit [42,43]. GenBank accession numbers are shown in Additonal file 5 Tables S5-S9. A similarity matrix of sequences was calculated in BioEdit.

## Authors' contributions

AJM participated in study design, generation of sequence alignments, sequence analysis, microarray analysis and in manuscript revisions. JAL participated in the study design and coordination, microarray analysis, and drafted the manuscript. All authors read and approved the final manuscript.

## Supplementary Material

Additional file 1 **"Variation in *S. aureus *surface proteins"**. shows the inter- lineage and intra-lineage proportions of variable sites in protein domains for 24 *Staphylococcus aureus *adhesins. Click here for file

Additional file 2 **"Variation in *S. aureus *secreted proteins involved in immune evasion"**. shows the inter- lineage and intra-lineage proportions of variable sites in protein domains for13 *Staphylococcus aureus *secreted proteins involved in immune evasion. Click here for file

Additional file 3 **"Distribution of domain variants of FnBPA across *S. aureus *lineages"**. shows the distribution of variants for each FnBPA domain is shown for15 *Staphylococcus aureus *clonal complex lineages. Click here for file

Additional file 4 **"Distribution of domain variants of Coa across *S. aureus *lineages"**. shows the distribution of variants for each Coa domain is shown for15 *Staphylococcus aureus *clonal complex lineages. Click here for file

Additional file 5 **"Variation in host factors of *S. aureus*"**. show the interspecies homology of host proteins in the form of a similarity matrix. Click here for file
