# Supplementary material for: Genetic variation in Staphylococcus aureus surface and immune evasion genes is lineage associated: implications for vaccine design and host-pathogen interactions
Source: BMC Microbiol. 2010 Jun 15;10:173. doi: 10.1186/1471-2180-10-173 (PMC2905362; doi:10.1186/1471-2180-10-173)
Supplement: Additional file 1 — "Variation in S. aureus surface proteins". shows the inter- lineage and intra-lineage proportions of variable sites in protein domains for 24 Staphylococcus aureus adhesins. [file 1471-2180-10-173-S1.DOC]

Table S1. Variation in *S. aureus* surface proteins.

| **Protein & function** | **Protein domains** | **Proportion of variable sites** | | | | | | | **Gene**  **Absence** | **Truncated protein** |
| --- | --- | --- | --- | --- | --- | --- | --- | --- | --- | --- |
|  |  |  |  |  |  |  |  |  |  |  |
|  |  | **Interlineage** | **Intralineage** | | | | | |  |  |
|  |  |  | ST5 | CC5 | ST8 | CC8 | ST30 | CC30 |  |  |
|  |  | n=58 | n=15 | n=17 | n=6 | n=7 | n=15 | n=18 |  |  |
| Aaa  (SAR0464) Autolysis & binds to Fg, Fn and Vn | Signal sequence (1-25) | 0.000 | 0.000 | 0.000 | 0.000 | 0.000 | 0.000 | 0.000 | EMRSA15(22) |  |
|  | Domain 1 (29-72) lysin domain | 0.000 | 0.000 | 0.000 | 0.000 | 0.000 | 0.000 | 0.000 |  |  |
|  | Domain 2 (93-136) lysin domain | 0.100 | 0.100 | 0.100 | 0.000 | 0.000 | 0.000 | 0.000 |  |  |
|  | Domain 3 (160-225) lysin domain | 0.123 | 0.100 | 0.100 | 0.000 | 0.000 | 0.000 | 0.000 |  |  |
|  | C terminus (226-335) | 0.018 | 0.009 | 0.009 | 0.000 | 0.000 | 0.000 | 0.000 |  |  |
| ClfA  (SAR0842)  Binds to Fg | Signal sequence (1-40) | 0.025 | 0.000 | 0.000 | 0.000 | 0.000 | 0.000 | 0.000 | A017934/97(30) |  |
|  | Fg Binding domain (41-560) | 0.200 | 0.002 | 0.004 | 0.003 | 0.003 | 0.000 | 0.000 |  |  |
|  | Repeat region (561-1023) | HIGH | YES | YES | YES | YES | YES | YES |  |  |
|  | Wall and membrane spanning domain (1024-1092) | 0.162 | 0.000 | 0.000 | 0.000 | 0.000 | 0.000 | 0.000 |  |  |
| ClfB  (SAR2709)  Binds to Fg and CK10 | Signal sequence (1-45) | 0.044 | 0.000 | 0.000 | 0.000 | 0.000 | 0.000 | 0.044 | S0385(398) | A9763(5) |
|  | Fg and CK10 binding domain (46-549) | 0.125 | 0.004 | 0.004 | 0.002 | 0.002 | 0.000 | 0.033 |  |  |
|  | Repeat region (550-926) | HIGH | YES | YES | YES | YES | YES | YES |  |  |
|  | Wall and membrane spanning domain (927-1000) | 0.068 | 0.000 | 0.000 | 0.000 | 0.000 | 0.000 | 0.000 |  |  |
| Cna  (SAR2774) Binds to collagen | Signal sequence (1-29) | 0.069 | - | - | - | - | 0.000 | 0.000 | CC5 CC8 CC10 TCH959(7) C160(30) C427(42) TCH130(72) RF122(151) S0385(398) M809(431) | A107934/97(30) WW2703/97(30) |
|  | N terminus (30-150) | 0.033 | - | - | - | - | 0.000 | 0.000 |  |  |
|  | Collagen binding domain (151-318) | 0.036 | - | - | - | - | 0.000 | 0.000 |  |  |
|  | Central region (319-524) | 0.044 | - | - | - | - | 0.000 | 0.000 |  |  |
|  | Repeat region (523-1095) | 0.049 | - | - | - | - | 0.012 | 0.012 |  |  |
|  | C-terminus (1096-1185) | 0.045 | - | - | - | - | 0.000 | 0.000 |  |  |
| Eap/Map (SAR2030)  Binds to extraceullar matrix Modulates cytokines | Signal sequence (1-50) | 0.220 | 0.000 | 0.000 | 0.000 | 0.000 | 0.000 | 0.000 | MSSA4776(1) JH1(5) FPR3757(8) A017934/97(30) WBG10049(30) | A6224(5) WW2703/97(30) RF122(151) |
|  | Repeat 1 (51-138) Vn, Fn | 0.205 | 0.000 | 0.000 | 0.000 | 0.000 | 0.000 | 0.000 |  |  |
|  | Repeat 2 (162-240) Vn, Fn, Fg, Cn-1, Tsp-1 | 0.166 | 0.013 | 0.013 | 0.000 | 0.000 | 0.000 | 0.000 |  |  |
|  | Repeat 3 (271-358) Vn, Fn, Fg, Cn-1, Tsp-1 | 0.241 | 0.011 | 0.011 | 0.000 | 0.000 | 0.000 | 0.000 |  |  |
|  | Repeat 4 (380-484) Vn, Fn, Fg, Cn-1, Tsp-1 | 0.365 | 0.000 | 0.000 | 0.000 | 0.000 | 0.000 | 0.000 |  |  |
|  | Repeat 5 (490-590) | 0.540 | - | - | 0.000 | 0.000 | 0.000 | 0.000 |  |  |
|  | Repeat 6 (591-697) | 0.330 | 0.009 | 0.009 | - | - | 0.000 | 0.009 |  |  |
| Ebh  (SAR1447)  Binds to Fn | Signal sequence (1-36) | 0.055 | 0.000 | 0.000 | 0.000 | 0.000 | 0.000 | 0.000 | MSSA476(1) | CC10 A5937(5) A9763(5) A9781(5) CF-Marseille(5) ED98(5) Mu3(5) Mu50(5) N315(5) A5948(8) A9765(8) Newman(8) A017934/97(30) 55/2053(30) 58-424(30) 65-1322(30) 68-397(30) Btn1260(30) C101(30) E1410(30) M876(30) M899(30) M1015(30) MN8(30) WW2703/97(30) WBG10049(30) C427(42) A9635(45) RF122(151) M809(431) |
|  | N terminus (37-2523) | 0.191 | 0.001 | 0.001 | 0.001 | 0.001 | 0.002 | 0.002 |  |  |
|  | Sugar binding domain (2524-3030) | 0.124 | 0.001 | 0.001 | 0.000 | 0.002 | 0.006 | 0.006 |  |  |
|  | Repeat region, albumin binding? (3031-8575) | 0.090 | 0.001 | 0.001 | 0.001 | 0.001 | 0.004 | 0.004 |  |  |
|  | C terminus (8576-10756) | 0.169 | 0.010 | 0.010 | 0.001 | 0.001 | 0.005 | 0.006 |  |  |
|  |  |  |  |  |  |  |  |  |  |  |
| EbpS  (SAR1489)  Binds to Eln | N terminus (1-13) | 0.000 | 0.000 | 0.000 | 0.000 | 0.000 | 0.000 | 0.000 |  | 58-424(5) |
|  | Elastin binding domain (14-34) | 0.050 | 0.000 | 0.000 | 0.000 | 0.000 | 0.000 | 0.000 |  |  |
|  | Extracellular (35-204) | 0.218 | 0.006 | 0.006 | 0.000 | 0.000 | 0.000 | 0.000 |  |  |
|  | Central region (205-341) | 0.132 | 0.000 | 0.000 | 0.000 | 0.000 | 0.000 | 0.000 |  |  |
|  | Extracellular (342-438) | 0.083 | 0.010 | 0.010 | 0.000 | 0.000 | 0.021 | 0.021 |  |  |
|  | LysM repeat (439-490) | 0.019 | 0.000 | 0.000 | 0.000 | 0.000 | 0.000 | 0.000 |  |  |
| FnBPA (SAR2580)  Binds to Fn, Fg and Eln | Signal sequence (1-37) | 0.189 | 0.000 | 0.000 | 0.000 | 0.000 | 0.000 | 0.027 |  | Newman(8) D139(10) |
|  | N terminus of variable region (38-195) | 0.299 | 0.000 | 0.000 | 0.000 | 0.000 | 0.000 | 0.000 |  |  |
|  | Fg and elastin binding domain (194-511) | 0.561 | 0.000 | 0.000 | 0.003 | 0.003 | 0.000 | 0.003 |  |  |
|  | Fn binding domain (512-885) | 0.139 | 0.032 | 0.032 | 0.016 | 0.016 | 0.008 | 0.008 |  |  |
|  | Repeat region (886-993) | HIGH | YES | YES | YES | YES | YES | YES |  |  |
|  | C terminus (994-1079) | 0.164 | 0.035 | 0.035 | 0.047 | 0.047 | 0.012 | 0.012 |  |  |
| FnBPB (SAN2290)  Binds to Fn and Eln | Signal sequence (1-36) | 0.083 | 0.000 | 0.000 | 0.000 | 0.000 | 0.000 | 0.000 | CC10  A5937(5) A6224(5) EMRSA15(22) A017934/97(30) Btn160(30) C160(30) MN8(30) MRSA252(30) WW2703/97(30) C427(42) TCH130(72) RF122(151) | Newman(8) ST398(398) |
|  | Variable region, binds Fn and Eln (37-540) | 0.431 | 0.002 | 0.002 | 0.004 | 0.004 | 0.000 | 0.000 |  |  |
|  | Repeat region (541-925) | HIGH | NO | NO | NO | NO | NO | NO |  |  |
|  | C terminus (925-1010) | 0.129 | 0.035 | 0.035 | 0.000 | 0.000 | 0.000 | 0.000 |  |  |
|  |  |  |  |  |  |  |  |  |  |  |
| IsaB  (SAR2717)  Elicits immune response, binding to nucleic acids | Signal sequence (1-36) | 0.389 | 0.000 | 0.000 | 0.000 | 0.000 | 0.000 | 0.000 |  |  |
|  | N terminus (37-100) | 0.151 | 0.027 | 0.027 | 0.000 | 0.000 | 0.000 | 0.000 |  |  |
|  | C terminus (101-176) | 0.107 | 0.000 | 0.000 | 0.000 | 0.000 | 0.013 | 0.026 |  |  |
|  |  |  |  |  |  |  |  |  |  |  |
| IsdA (SasE) (SAR1103) Binds to Fg, Fn, loricrin, involucrin, and CK10, Hb, transferring, heme | Signal sequence (1-46) | 0.022 | 0.000 | 0.000 | 0.000 | 0.000 | 0.000 | 0.000 |  |  |
|  | NEAT domain (69-177) ligand binding | 0.046 | 0.000 | 0.000 | 0.000 | 0.000 | 0.000 | 0.000 |  |  |
|  | C terminus (178-354) | 0.164 | 0.000 | 0.000 | 0.006 | 0.006 | 0.006 | 0.006 |  |  |
|  |  |  |  |  |  |  |  |  |  |  |
| IsdB (SasJ) (SAT1102) Binds to Hb, heme | N terminus (1-151) | 0.179 | 0.000 | 0.000 | 0.000 | 0.000 | 0.013 | 0.013 | TCH1516(8) |  |
|  | NEAT1 domain (152-272), Hb binding | 0.017 | 0.000 | 0.000 | 0.000 | 0.000 | 0.000 | 0.000 |  |  |
|  | NEAT 2 domain (349-476), heme binding | 0.047 | 0.000 | 0.000 | 0.000 | 0.000 | 0.000 | 0.008 |  |  |
|  | C terminus (477-653) | 0.153 | 0.000 | 0.000 | 0.000 | 0.000 | 0.000 | 0.000 |  |  |
| IsdH (HarA/SasI) (SAR1809) Binds to Hb, Hp | Signal sequence (1-35) | 0.086 | 0.000 | 0.000 | 0.000 | 0.000 | 0.000 | 0.000 |  | A9781(5) H19(10) A019734/97(30) Btn1260(30) C160(30) MN8(30) MRSA252(30) WW2703/97(30) RF122(151) |
|  | NEAT1 domain (101-232) Hb and Hp binding | 0.023 | 0.000 | 0.000 | 0.000 | 0.000 | 0.000 | 0.000 |  |  |
|  | NEAT2 domain (341-471) | 0.069 | 0.007 | 0.007 | 0.000 | 0.000 | 0.000 | 0.000 |  |  |
|  | NEAT3 domain (539-664) heme binding | 0.088 | 0.000 | 0.000 | 0.000 | 0.000 | 0.000 | 0.000 |  |  |
|  | C terminus (665-920) | 0.380 | 0.000 | 0.000 | 0.000 | 0.000 | 0.004 | 0.004 |  |  |
|  |  |  |  |  |  |  |  |  |  |  |
| SasB  (SAR2248)  No function reported | Signal sequence (1-39) | 0.026 | 0.000 | 0.000 | 0.000 | 0.000 | 0.000 | 0.000 | TCH1516(8) JKD6008(239) JKD6009(239) | CC1 FPR3757(8) A017934/97(30) Btn1260(30) M876(30) MRSA252(30) WW2703/97(30) C160(34) |
|  | N terminus (40-696) | 0.073 | 0.002 | 0.002 | 0.000 | 0.000 | 0.001 | 0.001 |  |  |
|  | 75 amino acid repeat region (697-1618) | 0.149 | 0.001 | 0.001 | 0.000 | 0.000 | 0.002 | 0.002 |  |  |
|  | C terminus (1619-2515) | 0.226 | 0.003 | 0.003 | 0.001 | 0.001 | 0.001 | 0.001 |  |  |
|  |  |  |  |  |  |  |  |  |  |  |
| SasC (Mrp) (SAR1841) Cell aggregation and biofilm formation | Signal sequence (1-37) | 0.027 | 0.000 | 0.000 | 0.000 | 0.000 | 0.000 | 0.000 | RF122(151) | A6624(5) Mu3(5) Mu50(5) A019734/97(30) Btn1260(30) WW2703/97 |
|  | N terminus (38-625) | 0.262 | 0.002 | 0.002 | 0.000 | 0.002 | 0.003 | 0.003 |  |  |
|  | 300 amino acid repeat region (626-1310) | 0.124 | 0.001 | 0.001 | 0.004 | 0.004 | 0.003 | 0.003 |  |  |
|  | C terminus (1311-2193) | 0.170 | 0.015 | 0.015 | 0.002 | 0.003 | 0.009 | 0.009 |  |  |
| SasD  (SAR0136) No function reported | Signal sequence (1-27) | 0.037 | 0.037 | 0.037 | 0.000 | 0.000 | 0.000 | 0.000 | H19(10) EMRSA15(22) LGA251(425) |  |
|  | N terminus (28-130) | 0.049 | 0.000 | 0.000 | 0.000 | 0.000 | 0.000 | 0.000 |  |  |
|  | C terminus (131-241) | 0.064 | 0.000 | 0.000 | 0.000 | 0.000 | 0.000 | 0.000 |  |  |
| SasF  (SAR2725) No function reported | Signal sequence (1-39) | 0.128 | 0.026 | 0.026 | 0.000 | 0.000 | 0.000 | 0.026 |  |  |
|  | N terminus (40-339) | 0.164 | 0.000 | 0.000 | 0.003 | 0.003 | 0.003 | 0.040 |  |  |
|  | C terminus (340-638) | 0.040 | 0.000 | 0.000 | 0.003 | 0.003 | 0.000 | 0.000 |  |  |
| SasG  (SAN2285) Adherence in nasal epithelial cells, autoaggregration | Signal sequence (1-50) | 0.000 | 0.000 | 0.000 | 0.000 | 0.000 | - | - | CC10 CC30 TCH959(8) C427(42) A9635(45) RF122(151) S0385(398) LGA251(425) M809(431) | A5937(5) Mu50(5) N315(5) A9765(8) FPR3757(8) TCH1516(8) |
|  | A domain functional (51-453) | 0.291 | 0.040 | 0.040 | 0.037 | 0.037 | - | - |  |  |
|  | B domain repeats (454-1530) | HIGH | YES | YES | YES | YES | - | - |  |  |
|  | C domain (1531-1633) | 0.039 | 0.000 | 0.000 | 0.000 | 0.000 | - | - |  |  |
|  |  |  |  |  |  |  |  |  |  |  |
| SasH (AdsA) (SAR0023) | Signal sequence (1-41) | 0.096 | 0.024 | 0.024 | 0.000 | 0.000 | 0.024 | 0.024 |  | A9765(8) |
|  | N terminus (42-415) | 0.166 | 0.005 | 0.005 | 0.043 | 0.043 | 0.030 | 0.030 |  |  |
|  | C terminus (416-788) | 0.126 | 0.000 | 0.000 | 0.000 | 0.000 | 0.003 | 0.027 |  |  |
| SasK  (SAN2381) No function reported | Signal sequence (1-26) | 0.038 | 0.000 | 0.000 | - | - | - | - | ST8 CC30 CC239 MSSA476(1) RF122(151) ST398(398) M809(431) C427(42) A9765(45) |  |
|  | N terminus (27-63) | 0.000 | 0.000 | 0.000 | - | - | - | - |  |  |
|  | 13 amino acid repeat region (64-121) | 0.122 | 0.018 | 0.018 | - | - | - | - |  |  |
|  | C terminus (122-215) | 0.140 | 0.011 | 0.011 | - | - | - | - |  |  |
|  |  |  |  |  |  |  |  |  |  |  |
| SdrC  (SAR0566) Adherence to extracellular matrix | Signal sequence (1-50) | 0.100 | 0.000 | 0.000 | 0.000 | 0.000 | 0.000 | 0.000 | 68-397(30) A017934/97(30) C160(30) M899(30) RF122(151) | A8117(5) |
|  | N terminus (51-495) Adherence? | 0.250 | 0.000 | 0.000 | 0.002 | 0.002 | 0.004 | 0.004 |  |  |
|  | B domain 1 (496-606) Ca^2+^ binding | 0.073 | 0.000 | 0.000 | 0.000 | 0.000 | 0.000 | 0.000 |  |  |
|  | B domain 2 (607-717) Ca^2+^ binding | 0.090 | 0.000 | 0.000 | 0.000 | 0.000 | 0.000 | 0.000 |  |  |
|  | Repeat region (718-935) | HIGH | YES | YES | YES | YES | YES | YES |  |  |
|  | C terminus (937-997) | 0.110 | 0.000 | 0.000 | 0.000 | 0.000 | 0.000 | 0.000 |  |  |
| SdrD  (SAN0520) Adherence to extracellular matrix | Signal sequence (1-35) | 0.056 | 0.000 | 0.000 | 0.000 | 0.000 | 0.028 | 0.028 | CC10 68-397(30) C160(30) MRSA252(30) C427(42) RF122(151) |  |
|  | A domain (36-575) Adherence? | 0.396 | 0.002 | 0.002 | 0.122 | 0.122 | 0.000 | 0.000 |  |  |
|  | B domain 1 (576-684) Ca^2+^ binding | 0.196 | 0.009 | 0.009 | 0.000 | 0.000 | 0.000 | 0.000 |  |  |
|  | B domain 2 (685-795) Ca^2+^ binding | 0.127 | 0.009 | 0.009 | 0.000 | 0.000 | 0.000 | 0.000 |  |  |
|  | B domain 3 (796-903) Ca^2+^ binding | 0.047 | 0.000 | 0.000 | 0.000 | 0.000 | 0.000 | 0.000 |  |  |
|  | B domain 4 (904-1011) Ca^2+^ binding | 0.009 | 0.000 | 0.000 | 0.000 | 0.000 | 0.000 | 0.000 |  |  |
|  | B domain 5 (1012-1128) Ca^2+^ binding | 0.129 | 0.000 | 0.000 | 0.000 | 0.000 | 0.000 | 0.000 |  |  |
|  | Ser-Asp repeat region (1129-1244) | HIGH | YES | YES | YES | YES | YES | YES |  |  |
|  | C terminus (1345-1405) | 0.233 | 0.016 | 0.016 | 0.150 | 0.150 | 0.000 | 0.000 |  |  |
| SdrE  (SAR0567) Adherence to extracellular matrix and platelet aggregation | Signal sequence (1-52) | 0.000 | 0.000 | 0.000 | 0.000 | 0.000 | 0.000 | 0.000 | CC10 CF-Marseille(5) TCH959(7) A9765(8) NCTC 8325(8) 68-397(30) A9635(45)  ST398(398) RF122(151) | A5937(5) |
|  | A domain Ligand binding (53-605) | 0.270 | 0.000 | 0.000 | 0.000 | 0.002 | 0.004 | 0.004 |  |  |
|  | B domain 1 (606-719) | 0.044 | 0.008 | 0.008 | 0.000 | 0.000 | 0.000 | 0.000 |  |  |
|  | B domain 2 (720-829) | 0.028 | 0.000 | 0.000 | 0.009 | 0.009 | 0.000 | 0.000 |  |  |
|  | B domain 3 (830-939) | 0.046 | 0.009 | 0.009 | 0.000 | 0.000 | 0.009 | 0.009 |  |  |
|  | Ser-Asp repeat region (940-1119) | HIGH | YES | YES | YES | YES | YES | YES |  |  |
|  | C terminus (1120-1179) | 0.017 | 0.000 | 0.000 | 0.000 | 0.000 | 0.000 | 0.000 |  |  |
| Spa  (SAR0114) Binds to IgG, TNFR1 and von Willebrand factor | Signal sequence (1-48) | 0.000 | 0.000 | 0.000 | 0.000 | 0.000 | 0.000 | 0.000 |  | A8115(5) ED98(5) RF122(151) |
|  | IgG binding domain (49-339) | 0.124 | 0.006 | 0.006 | 0.037 | 0.037 | 0.048 | 0.059 |  |  |
|  | X region (340-441) | HIGH | YES | YES | YES | YES | YES | YES |  |  |
|  | C terminus (442-546) | 0.029 | 0.000 | 0.000 | 0.000 | 0.000 | 0.000 | 0.000 |  |  |
| SraP (SasA) (SAR2734) Binding to platelets | Signal sequence (1-90) | 0.044 | 0.000 | 0.000 | 0.000 | 0.000 | 0.000 | 0.022 | S0385 (398) |  |
|  | A region N1 (91-244) binding | 0.136 | 0.000 | 0.000 | 0.000 | 0.000 | 0.000 | 0.078 |  |  |
|  | A region N2 (245-476) binding | 0.078 | 0.000 | 0.000 | 0.004 | 0.004 | 0.004 | 0.030 |  |  |
|  | A region N3 (477-575) binding | 0.010 | 0.000 | 0.000 | 0.000 | 0.000 | 0.000 | 0.010 |  |  |
|  | Central & Repeat region (576-2201) | HIGH | YES | YES | YES | YES | YES | YES |  |  |
|  | C terminus (2202-2290) | 0.359 | 0.000 | 0.000 | 0.000 | 0.000 | 0.000 | 0.000 |  |  |

The inter-lineage and intra-lineage proportions of variable sites in protein domains are shown for 13 *Staphylococcus aureus* secreted proteins involve din immune evasion. For each *S. aureus* secreted protein, pseudonyms, function and reference gene number from the MRSA252 (SAR----) or N315 (SAN----) genomes is shown. Secreted proteins are split into protein domains if domains have been characterised or N- and C-terminal regions if protein domains are uncharacterised, and appropriate references are listed. Inter-lineage variation is shown for individual protein domains/regions based on 58 sequences from 15 different clonal complexes (CCs) and 20 different sequence types (STs). Intra-lineage variation is shown for individual protein domains/region for CC5, CC8, CC30, ST5, ST8 and ST30. Levels of proportions of variation are coloured differentially; no variation (white), 0.001 to 0.100 proportion of variable sites (light grey), 0.101 to 0.200 proportion of variable sites (grey), 0.201 to 0.300 proportion of variable sites (dark grey), and a proportion of variable sites that is greater than 0.300 (black). Absence of a gene from a genome or truncation of a protein product is shown in the absent and truncated columns respectively.
