# Supplementary material for: Genetic variation in Staphylococcus aureus surface and immune evasion genes is lineage associated: implications for vaccine design and host-pathogen interactions
Source: BMC Microbiol. 2010 Jun 15;10:173. doi: 10.1186/1471-2180-10-173 (PMC2905362; doi:10.1186/1471-2180-10-173)
Supplement: Additional file 2 — "Variation in S. aureus secreted proteins involved in immune evasion". shows the inter- lineage and intra-lineage proportions of variable sites in protein domains for13 Staphylococcus aureus secreted proteins involved in immune evasion. [file 1471-2180-10-173-S2.DOC]

Table S2. Variation in *S. aureus* secreted proteins involved in immune evasion.

| **Protein & function** | **Protein domains** | **Proportion of variable sites** | | | | | | | **Gene Absence** | **Truncated protein** |
| --- | --- | --- | --- | --- | --- | --- | --- | --- | --- | --- |
|  |  |  |  |  |  |  |  |  |  |  |
|  |  | **Interlineage** | **Intralineage** | | | | | |  |  |
|  |  |  | ST5 | CC5 | ST8 | CC8 | ST30 | CC30 |  |  |
|  |  | n=58 | n=15 | n=17 | n=6 | n=7 | n=15 | n=17 |  |  |
| Coa (SAR0222) | Signal sequence (1-26) | 0.000 | 0.000 | 0.000 | 0.000 | 0.000 | 0.000 | 0.000 |  | A8115 (5) |
|  | Domain 1 (27-171) PT binding | 0.815 | 0.000 | 0.000 | 0.000 | 0.000 | 0.000 | 0.000 |  |  |
|  | Domain 2 (172-304) PT binding | 0.696 | 0.000 | 0.000 | 0.000 | 0.000 | 0.008 | 0.008 |  |  |
|  | Central region (305-523) | 0.298 | 0.000 | 0.000 | 0.000 | 0.000 | 0.000 | 0.000 |  |  |
|  | Repeat region (524-728) | 0.221 | 0.064 | 0.064 | 0.005 | 0.005 | 0.039 | 0.039 |  |  |
|  | C terminus (728-774) | 0.587 | 0.052 | 0.052 | 0.000 | 0.000 | 0.000 | 0.000 |  |  |
| Ecb  (SAR1127) Blocks complement | Signal sequence (1-29) | 0.034 | 0.000 | 0.000 | 0.000 | 0.000 | 0.000 | 0.000 |  |  |
|  | N terminus (30-69) | 0.267 | 0.000 | 0.000 | 0.000 | 0.000 | 0.000 | 0.000 |  |  |
|  | C terminus (70-110) | 0.000 | 0.000 | 0.000 | 0.000 | 0.000 | 0.000 | 0.000 |  |  |
| Efb (Fib) (SAR1130) Binds to FG Complement inhibition | Signal sequence (1-29) | 0.207 | 0.000 | 0.000 | 0.000 | 0.000 | 0.000 | 0.000 |  | 65-1322(30) |
|  | N terminus (30-45) | 0.133 | 0.000 | 0.000 | 0.000 | 0.000 | 0.000 | 0.000 |  |  |
|  | Repeat region (46-97) FG binding | 0.176 | 0.000 | 0.000 | 0.000 | 0.000 | 0.000 | 0.000 |  |  |
|  | C terminus (97-168) C3b binding | 0.183 | 0.000 | 0.000 | 0.000 | 0.000 | 0.014 | 0.028 |  |  |
| Emp  (SAR0845) Binds to extracellular matrix | Signal sequence (1-26) | 0.154 | 0.000 | 0.000 | 0.000 | 0.000 | 0.000 | 0.000 |  |  |
|  | N terminus (27-183) | 0.186 | 0.000 | 0.000 | 0.000 | 0.000 | 0.000 | 0.000 |  |  |
|  | C terminus (184-340) | 0.294 | 0.006 | 0.006 | 0.000 | 0.000 | 0.006 | 0.006 |  |  |
|  |  |  |  |  |  |  |  |  |  |  |
| EsaC  (SAN0277) Secreted, virulence factor, unknown function | N terminus (1-65) | 0.031 | 0.000 | 0.000 | 0.000 | 0.000 | - | - | CC10 CC30 CC239 EMRSA15(22) C427(42) ST398(398) LGA251(425) M809(431) |  |
|  | C terminus (66-130) | 0.062 | 0.000 | 0.000 | 0.000 | 0.000 | - | - |  |  |
|  |  |  |  |  |  |  |  |  |  |  |
| EsxA (SAR0279) | N terminus (1-49) | 0.020 | 0.000 | 0.000 | 0.000 | 0.000 | 0.000 | 0.000 |  |  |
|  | C terminus (50-98) | 0.000 | 0.000 | 0.000 | 0.000 | 0.000 | 0.000 | 0.000 |  |  |
| EssC (SAR0284) Required for secretion of EsxA | N terminus (1-651) | 0.043 | 0.000 | 0.000 | 0.000 | 0.000 | 0.005 | 0.006 |  | H19(10) C427(42) |
|  | FtsK domain 1 (652-846) | 0.031 | 0.000 | 0.000 | 0.000 | 0.000 | 0.000 | 0.000 |  |  |
|  | Central region (847-996) | 0.007 | 0.000 | 0.000 | 0.000 | 0.000 | 0.000 | 0.000 |  |  |
|  | FtsK domain 2 (997-1183) | 0.290 | 0.000 | 0.000 | 0.000 | 0.000 | 0.000 | 0.000 |  |  |
|  | C terminus (1184-1483) | 0.796 | 0.000 | 0.000 | 0.000 | 0.000 | 0.000 | 0.003 |  |  |
| FLIPr  (SAN1001) Binds to FPRL1 and FPR | Signal sequence (1-17) | 0.118 | 0.000 | 0.000 | 0.000 | 0.000 | - | - | CC30 C427(42) A9635(45) ST398(398) M809(431) |  |
|  | N terminus (18-75) | 0.473 | 0.000 | 0.000 | 0.000 | 0.000 | - | - |  |  |
|  | C terminus (76-133) | 0.351 | 0.000 | 0.000 | 0.017 | 0.017 | - | - |  |  |
|  |  |  |  |  |  |  |  |  |  |  |
| FLIPr-like (SAR1128) Binds to FPRL1 and FPR | Signal sequence (1-21) | 0.238 | - | - | - | - | 0.143 | 0.143 | Present: CC30 C427(42) A9635(45) ST398(398) M809(431) | 55/2053(30) 58-424(30) 65-1322(30) 68/397(30) C101(30) E1410(30) M876(30) M899(30) M1015(30) M809(431) |
|  | N terminus (22-78) | 0.018 | - | - | - | - | 0.018 | 0.018 |  |  |
|  | C terminus (79-135) | 0.482 | - | - | - | - | 0.018 | 0.018 |  |  |
|  |  |  |  |  |  |  |  |  |  |  |
| Sbi (SAR2508) Binds to IgG Alternate pathway modulation | Signal sequence (1-26) | 0.000 | 0.000 | 0.000 | 0.000 | 0.000 | 0.000 | 0.000 |  |  |
|  | N terminus (27-40) | 0.077 | 0.000 | 0.000 | 0.000 | 0.000 | 0.000 | 0.000 |  |  |
|  | Domain I (41-95) Ig binding domain | 0.018 | 0.000 | 0.000 | 0.000 | 0.000 | 0.000 | 0.000 |  |  |
|  | Domain II (103-153) Ig binding domain | 0.040 | 0.000 | 0.000 | 0.020 | 0.020 | 0.000 | 0.000 |  |  |
|  | Domain III (154-196) C3 binding domain | 0.000 | 0.000 | 0.000 | 0.000 | 0.000 | 0.000 | 0.000 |  |  |
|  | Domain IV (197-253) C3 binding domain | 0.125 | 0.000 | 0.000 | 0.000 | 0.000 | 0.000 | 0.000 |  |  |
|  | C terminus (254-457) | 0.251 | 0.005 | 0.005 | 0.000 | 0.000 | 0.000 | 0.000 |  |  |
| SCIN-B (SAN1004) Complement inhibition, stabilises C4b2a | Signal sequence (1-17) | 0.000 | 0.000 | 0.000 | 0.000 | 0.000 | - | - | CC30 EMRSA15(22)  ST398(398) |  |
|  | N terminus (18-67) | 0.163 | 0.000 | 0.000 | 0.000 | 0.000 | - | - |  |  |
|  | C terminus (68-117) | 0.142 | 0.000 | 0.000 | 0.000 | 0.000 | - | - |  |  |
| SCIN-C  (SAR1131) Complement inhibition | Signal sequence (1-22) | 0.091 | - | - | - | - | 0.000 | 0.000 | Present: CC30 EMRSA15(22)  ST398(398) |  |
|  | N terminus (23-70) | 0.234 | - | - | - | - | 0.000 | 0.000 |  |  |
|  | C terminus (71-117) | 0.196 | - | - | - | - | 0.000 | 0.000 |  |  |
| VwBP  (SAR0843) Binds to vWF Interacts with ProT | Signal sequence (1-26) | 0.038 | 0.000 | 0.000 | 0.000 | 0.000 | 0.000 | 0.000 |  | MW2(1) TCH70(1) A017934/97(30) Btn1260(30) C160(30) MN8(30) MRSA252(30) WBG10049(30) WW2703/97(30) |
|  | PT activation domain (27-297) | 0.866 | 0.011 | 0.011 | 0.000 | 0.000 | 0.019 | 0.019 |  |  |
|  | vWF binding domain (363-388) | 0.280 | 0.000 | 0.000 | 0.000 | 0.000 | 0.000 | 0.000 |  |  |
|  | C terminus (389-517) | 0.172 | 0.000 | 0.000 | 0.008 | 0.008 | 0.000 | 0.000 |  |  |
|  |  |  |  |  |  |  |  |  |  |  |

The inter-lineage and intra-lineage proportions of variable sites in protein domains are shown for 13 *Staphylococcus aureus* secreted proteins involve din immune evasion. For each *S. aureus* secreted protein, pseudonyms, function and reference gene number from the MRSA252 (SAR----) or N315 (SAN----) genomes is shown. Secreted proteins are split into protein domains if domains have been characterised or N- and C-terminal regions if protein domains are uncharacterised, and appropriate references are listed. Inter-lineage variation is shown for individual protein domains/regions based on 58 sequences from 15 different clonal complexes (CCs) and 20 different sequence types (STs). Intra-lineage variation is shown for individual protein domains/region for CC5, CC8, CC30, ST5, ST8 and ST30. Levels of proportions of variation are coloured differentially; no variation (white), 0.001 to 0.100 proportion of variable sites (light grey), 0.101 to 0.200 proportion of variable sites (grey), 0.201 to 0.300 proportion of variable sites (dark grey), and a proportion of variable sites that is greater than 0.300 (black). Absence of a gene from a genome or truncation of a protein product is shown in the absent and truncated columns respectively.
