# Supplementary material for: Genetic variation in Staphylococcus aureus surface and immune evasion genes is lineage associated: implications for vaccine design and host-pathogen interactions
Source: BMC Microbiol. 2010 Jun 15;10:173. doi: 10.1186/1471-2180-10-173 (PMC2905362; doi:10.1186/1471-2180-10-173)
Supplement: Additional file 3 — "Distribution of domain variants of FnBPA across S. aureus lineages". shows the distribution of variants for each FnBPA domain is shown for15 Staphylococcus aureus clonal complex lineages. [file 1471-2180-10-173-S3.DOC]

Table S3. Distribution of domain variants of FnBPA across *S. aureus* lineages

| **FnBPA domain** | **Lineage** | | | | | | | | | | | | | | |
| --- | --- | --- | --- | --- | --- | --- | --- | --- | --- | --- | --- | --- | --- | --- | --- |
|  | 1 | 5 | 7 | 8 | 10 | 22 | 30 | 42 | 45 | 72 | 151 | 239 | 398 | 425 | 431 |
| Signal sequence (1-37) | A, 2 | A, 1 | A | A | A, 2 | A, 2 | A | A | A, 1 | A, 1 | A, 1 | A | A, 2 | A, 1 | A |
| N terminus of variable region (38-195) | J | B | G | C | D | J | A | A | K | H | E | C | L | F | A |
| FG and ELN binding domain (194-511) | B | B | E | C | D | B | A | A | B, 1 | C, 8 | D, 3 | C | F | E | A |
| FN-1 binding domain (512-885) | A | B | C, 2 | C | A, 2 | D | A | A | E | C, 5 | A, 1 | C | C, 6 | A, 2 | A |
| Repeat region (886-993) | - | - | - | - | - | - | - | - | - | - | - | - | - | - | - |
| C terminus (994-1079) | A | A | A | A | A | A | A | A | A | A | A | A | A | A | A |

The distribution of variants for each FnBPA domain is shown for 15 *Staphylococcus aureus* clonal complex lineages. For each FnBPA protein domain, major domain variants are listed as letters (A to L), and a minor domain variant and the number of substitutions from a reference is listed in numbers (1 means that there is 1 amino acid substitution compared to the reference). The repeat region is highly variable and is unqiue between strains and lineages. A FASTA file of the FnBPA alignment is available on request from the authors.
