# Supplementary material for: Genetic variation in Staphylococcus aureus surface and immune evasion genes is lineage associated: implications for vaccine design and host-pathogen interactions
Source: BMC Microbiol. 2010 Jun 15;10:173. doi: 10.1186/1471-2180-10-173 (PMC2905362; doi:10.1186/1471-2180-10-173)
Supplement: Additional file 4 — "Distribution of domain variants of Coa across S. aureus lineages". shows the distribution of variants for each Coa domain is shown for15 Staphylococcus aureus clonal complex lineages. [file 1471-2180-10-173-S4.DOC]

Table S4. Distribution of domain variants of Coa across *S. aureus* lineages

| **Coa domain** | **Lineage** | | | | | | | | | | | | | | |
| --- | --- | --- | --- | --- | --- | --- | --- | --- | --- | --- | --- | --- | --- | --- | --- |
|  | 1 | 5 | 7 | 8 | 10 | 22 | 30 | 42 | 45 | 72 | 151 | 239 | 398 | 425 | 431 |
| Signal sequence (1-26) | A | A | A | A | A | A | A | A | A | A | A | A | A | A | A |
| Domain 1 (27-171) PT binding | F | A | C, 1 | C | D | E | B | B | F | G | H | B | F | A | B |
| Domain 2 (172-304) PT binding | F | A | C | C | D | E | B | B | F | G | H | B | F | A, 10 | B |
| Central region (305-523) | F | A | K | C | D | E | B | B, 1 | I | G | H | B | I | J | B |
| Repeat region (524-728) | - | - | - | - | - | - | - | - | - | - | - | - | - | - | - |
| C terminus (728-774) | E | A | B | C | B | F | B | B | G | H | I | B | D | D | B |

The distribution of variants for each Coa domain is shown for 15 *Staphylococcus aureus* clonal complex lineages. For each Coa protein domain, major domain variants are listed as letters (A to K), and a minor domain variant and the number of substitutions from a reference is listed in numbers (1 means that there is 1 amino acid substitution compared to the reference). The repeat region is highly variable and is unique between strains and lineages. A FASTA file of the Coa alignment is available on request from the authors.
