# Supplementary material for: Genetic variation in Staphylococcus aureus surface and immune evasion genes is lineage associated: implications for vaccine design and host-pathogen interactions
Source: BMC Microbiol. 2010 Jun 15;10:173. doi: 10.1186/1471-2180-10-173 (PMC2905362; doi:10.1186/1471-2180-10-173)
Supplement: Additional file 5 — "Variation in host factors of S. aureus". show the interspecies homology of host proteins in the form of a similarity matrix. [file 1471-2180-10-173-S5.DOC]

Table S5. Interspecies homology of FGB

|  | **Human** | **Chimpanzee** | **Mouse** | **Rat** | **Dog** | **Cow** | **Horse** | **Chicken** |
| --- | --- | --- | --- | --- | --- | --- | --- | --- |
| **Human**  **GenBank:NM_005141** | - | 0.987 | 0.818 | 0.792 | 0.846 | 0.797 | 0.813 | 0.607 |
| **Chimpanzee**  **GenBank:XM_001137030** | 0.987 | - | 0.810 | 0.785 | 0.838 | 0.792 | 0.811 | 0.600 |
| **Mouse**  **GenBank:NM_181849** | 0.818 | 0.81 | - | 0.896 | 0.783 | 0.776 | 0.754 | 0.594 |
| **Rat**  **GenBank:NM_020071** | 0.792 | 0.785 | 0.896 | - | 0.758 | 0.753 | 0.738 | 0.588 |
| **Dog**  **GenBank:XM_848645** | 0.843 | 0.838 | 0.783 | 0.758 | - | 0.807 | 0.821 | 0.622 |
| **Cow**  **GenBank:XM_587666** | 0.797 | 0.792 | 0.776 | 0.753 | 0.807 | - | 0.756 | 0.598 |
| **Horse**  **GenBank:XM_001500955** | 0.813 | 0.811 | 0.754 | 0.738 | 0.821 | 0.756 | - | 0.600 |
| **Chicken**  **GenBank:XM_420369** | 0.607 | 0.600 | 0.594 | 0.588 | 0.622 | 0.598 | 0.600 | - |

A similarity matrix between FGB protein sequences of two host species is shown. Identical sequences would score as 1.000 and two sequences that share no common sites would score as 0.000. GenBank accession numbers for each sequence used in the comparison are shown.

Table S6. Interspecies homology of FGG

|  | **Human** | **Chimpanzee** | **Mouse** | **Rat** | **Dog** | **Cow** | **Horse** |
| --- | --- | --- | --- | --- | --- | --- | --- |
| **Human**  **GenBank:NM_000509** | - | 0.922 | 0.821 | 0.814 | 0.852 | 0.837 | 08.57 |
| **Chimpanzee**  **GenBank:XM_001138780** | 0.992 | - | 0.821 | 0.812 | 0.854 | 0.837 | 0.856 |
| **Mouse**  **GenBank:NM_133862** | 0.821 | 0.821 | - | 0.907 | 0.782 | 0.770 | 0.774 |
| **Rat**  **GenBank:NM_012559** | 0.814 | 0.812 | 0.907 | - | 0.781 | 0.752 | 0.765 |
| **Dog**  **GenBank:XM_848659** | 0.852 | 0.854 | 0.782 | 0.781 | - | 0.837 | 0.857 |
| **Cow**  **GenBank:NM_173911** | 0.837 | 0.837 | 0.770 | 0.752 | 0.837 | - | 0.830 |
| **Horse**  **GenBank:XM_001914798** | 0.857 | 0.856 | 0.774 | 0.765 | 0.857 | 0.830 | - |

A similarity matrix between FGG protein sequences of two host species is shown. Identical sequences would score as 1.000 and two sequences that share no common sites would score as 0.000. GenBank accession numbers for each sequence used in the comparison are shown.

Table S7. Interspecies homology of FN-1

|  | **Human** | **Chimpanzee** | **Mouse** | **Rat** | **Cow** | **Horse** |
| --- | --- | --- | --- | --- | --- | --- |
| **Human**  **GenBank:NM_212476** | - | 0.992 | 0.864 | 0.865 | 0.897 | 0.905 |
| **Chimpanzee**  **GenBank:XM_516072** | 0.992 | - | 0.864 | 0.864 | 0.896 | 0.905 |
| **Mouse**  **GenBank:NM_010233** | 0.864 | 0.864 | - | 0.945 | 0.855 | 0.858 |
| **Rat**  **GenBank:CH474044** | 0.865 | 0.864 | 0.945 | - | 0.855 | 0.860 |
| **Cow**  **GenBank:XP_879240** | 0.897 | 0.896 | 0.855 | 0.855 | - | 0.907 |
| **Horse**  **GenBank:XM_001489104** | 0.905 | 0.905 | 0.858 | 0.860 | 0.907 | - |

A similarity matrix between FN-1 protein sequences of two host species is shown. Identical sequences would score as 1.000 and two sequences that share no common sites would score as 0.000. GenBank accession numbers for each sequence used in the comparison are shown.

Table S8. Interspecies homology of PT

|  | **Human** | **Orangutan** | **Mouse** | **Rat** | **Cow** | **Pig** | **Horse** | **Chicken** |
| --- | --- | --- | --- | --- | --- | --- | --- | --- |
| **Human**  **GenBank:NM_000552** | - | 0.948 | 0.809 | 0.791 | 0.812 | 0.828 | 0.815 | 0.609 |
| **Orangutan**  **GenBank:CR861038** | 0.948 | - | 0.781 | 0.768 | 0.779 | 0.799 | 0.794 | 0.596 |
| **Mouse**  **GenBank:NM_010168** | 0.809 | 0.781 | - | 0.933 | 0.769 | 0.796 | 0.782 | 0.629 |
| **Rat**  **GenBank:X52835** | 0.791 | 0.768 | 0.933 | - | 0.753 | 0.778 | 0.770 | 0.627 |
| **Cow**  **GenBank:NM_173877** | 0.812 | 0.779 | 0.769 | 0.753 | - | 0.849 | 0.788 | 0.616 |
| **Pig**  **GenBank:DQ530370** | 0.828 | 0.799 | 0.796 | 0.778 | 0.849 | - | 0.829 | 0.616 |
| **Horse**  **GenBank:XM_001490842** | 0.815 | 0.794 | 0.782 | 0.770 | 0.788 | 0.829 | - | 0.614 |
| **Chicken**  **GenBank:NM_204605** | 0.609 | 0.596 | 0.629 | 0.627 | 0.616 | 0.616 | 0.614 | - |

A similarity matrix between PT protein sequences of two host species is shown. Identical sequences would score as 1.000 and two sequences that share no common sites would score as 0.000. GenBank accession numbers for each sequence used in the comparison are shown.

Table S9. Interspecies homology of vWF

|  | **Human** | **Chimpanzee** | **Mouse** | **Rat** | **Dog** | **Cow** | **Pig** | **Chicken** |
| --- | --- | --- | --- | --- | --- | --- | --- | --- |
| **Human**  **GenBank:NM_000552** | - | 0.833 | 0.990 | 0.766 | 0.862 | 0.810 | 0.559 | 0.745 |
| **Chimpanzee**  **GenBank:XM_508945** | 0.833 | - | 0.832 | 0.737 | 0.829 | 0.911 | 0.551 | 0.720 |
| **Mouse**  **GenBank:NM_011708** | 0.990 | 0.832 | - | 0.766 | 0.862 | 0.808 | 0.560 | 0.745 |
| **Rat**  **GenBank:XM_342759** | 0.766 | 0.737 | 0.766 | - | 0.765 | 0.733 | 0.513 | 0.752 |
| **Dog**  **GenBank:NM_001002932** | 0.862 | 0.829 | 0.862 | 0.765 | - | 0.809 | 0.558 | 0.746 |
| **Cow**  **GenBank:XM_584169** | 0.810 | 0.911 | 0.808 | 0.733 | 0.809 | - | 0.545 | 0.713 |
| **Pig**  **GenBank:AF052036** | 0.559 | 0.551 | 0.560 | 0.513 | 0.558 | 0.545 | - | 0.491 |
| **Chicken**  **GenBank:XM_417223** | 0.745 | 0.720 | 0.745 | 0.752 | 0.746 | 0.713 | 0.491 | - |

A similarity matrix between vWF protein sequences of two host species is shown. Identical sequences would score as 1.000 and two sequences that share no common sites would score as 0.000. GenBank accession numbers for each sequence used in the comparison are shown.
